# Unraveling the Inner Electronic Structure of Chromium-Oxide Films by Probing the Layer-by-Layer Evolution of Their Workfunction

**DOI:** 10.1021/acs.jpclett.5c01617

**Published:** 2025-07-07

**Authors:** Ghada Missaoui, Jacek Goniakowski, Claudine Noguera, Niklas Nilius

**Affiliations:** † 232751 Carl von Ossietzky Universität Oldenburg, Institut für Physik, D-26111 Oldenburg, Germany; ‡ 84219 CNRS-Sorbonne Université, Institut des Nanosciences de Paris, UMR 7588, F-75005 Paris, France

## Abstract

Low-temperature tunneling spectroscopy has been employed to probe the workfunction ϕ of atomically flat Cr-oxide single- and double-stack films grown on Pt(111). While the single-stack Cr_3_O_6_ trilayer has a ϕ value of ∼7.0 eV, it decreases to ∼5.0 eV for a Cr_6_O_11_ double-stack. The charge redistribution underlying this workfunction drop has been analyzed by density functional theory. The remarkably high ϕ value of the Cr_3_O_6_/Pt trilayer, making it a highly electronegative substrate, originates from a massive electron transfer from the Pt(111) into empty Cr-states that reduces 2/3 of the oxide cations from their formal 4+ to a 3+ charge state. The negative surface dipole diminishes upon growing a Cr–O honeycomb layer on top of the trilayer, forming a Cr_6_O_11_ double-stack. The adlayer acts as electron donor, enabling the charge transfer from the Pt support to decrease substantially. The charge redistribution not only triggers the detected workfunction drop but also stabilizes the double-stack with respect to a single-stack geometry. A comparison of the observed CrO_*x*_/Pt behavior to that of hypothetical double-stack films, made of an interfacial TMO_2_ trilayer (TM = Ti, V, Mn, Fe) and a capping Cr–O honeycomb plane, allows us to correlate the charge transfer between the individual oxide layers and the substrate to the overall stability of the metal-oxide system.

## Graphical abstract

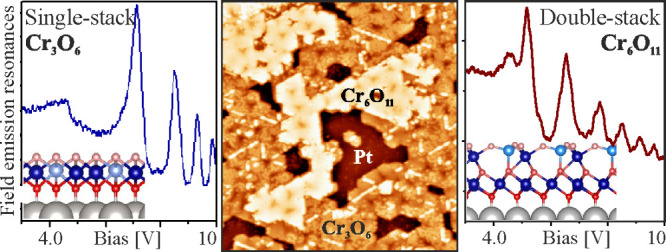

Understanding metal-oxide interactions is pivotal for the rational design of supported and inverse metal catalysts, where either small metal islands sit on bulk-oxide samples or vice versa.^1–3^ The unique electronic structure of ultrasmall metal clusters on dielectric supports was systematically explored in the past, revealing unique quantization phenomena, such as bandgap openings, formation of quantum-well states (QWSs) and electron-localization at low-coordinated sites.^4–6^ Spatially confined oxide islands and thin films grown on metal supports exhibit a similarly complex inner electronic structure, which has however attracted much less attention.^7,8^ While QWSs play a minor role in oxide nanostructures, given the absence of free electrons, several other factors introduce highly variable electronic properties in the individual oxide layers. (i) Band offsets and workfunction differences trigger an electron transfer between oxide and metal support.^9,10^ (ii) Depending on the oxygen affinity of the substrate, the availability of interfacial oxygen varies considerably.^11^ (iii) The lattice structure in multistack films often changes in a layer-by-layer fashion, as interface layers tend to adopt a favorable registry with the metal below while subsequent layers evolve toward the bulk oxide structure.^12^ And (iv), a layer-specific chemical composition emerges especially in ternary oxide films, as a means to minimize strain effects.^13,14^ In fact, structural modifications in individual layers of a multistack film are more the rule than the exception and well documented for many cases, e.g. Al_10_O_13_ bilayers on NiAl(110),^15^ MoO_3_ films on Pd(100),^16^ as well as SiFeO_4_/Ru­(0001),^13^ MnWO_*x*_ and FeWO_4_ on Pd(100).^14^ Only a few rocksalt oxides adopt their bulk lattice structure directly from the first layer on, yet with sometimes pronounced relaxation effects.^17^

The structural evolution in adjacent layers of a multistack oxide film is always accompanied by changes of its electronic response, resulting in a unique charge and dipole distribution along its growth direction.^18^ These layer-by-layer electronic modulations directly affect the film’s functionality, i.e., its workfunction, adsorption behavior, magnetic, ferroelectric and even optical properties.^19^ The underlying mechanisms are often inaccessible to direct measurements and can be retrieved only by theoretical means. In fact, most experimental techniques are only sensitive to collective quantities, e.g. the overall workfunction, that arise from the electron distribution across all layers of the oxide stack.

The present study aims at overcoming this information deficit by profiling the layer-specific electronic structure of Cr-oxide thin films grown on Pt(111). It takes advantage of the fact that this multistack oxide can be assembled in a layer-by-layer fashion and its properties can be sequentially probed as a function of thickness. As discussed earlier,^20^ the interface stack hereby takes the form of an O–Cr–O trilayer that gets capped by a Cr_2_O_3_ honeycomb (hc) plane at higher Cr coverage. For each stack, the local workfunction ϕ was determined with scanning tunneling microscopy (STM) combined with effective barrier-height, d­(*lnI*)/d*z*, and field-emission resonance (FER) spectroscopy.^21,22^ The detected workfunction evolution indicates distinct charge-transfer phenomena between the subsequent oxide layers and the metal below, with high and low ϕ values implying electron flow toward or away from the surface, respectively.^10^ Accompanying DFT calculations confirm a charge flow between the oxide layers and the Pt support and demonstrate its importance for the overall film stability. A theoretical comparison of the CrO_*x*_/Pt­(111) system with similar films, comprising a TiO_2_, VO_2_, MnO_2_ or FeO_2_ interface trilayer and a Cr_2_O_3_ top plane, gives further insights on how the inner electronic structure of a multistack oxide film governs its overall stability.

Single-stack Cr-oxide films grow in triangular domains of ∼ 100 Å edge length and 2.8 Å apparent height (Figure 1a). The unique domain structure is enforced by the lattice mismatch with the Pt(111) support and competes with a network of dislocation lines as an alternative means to reduce strain effects. Two lattice configurations are revealed in atomically resolved STM images (Figure 1b,c). At a bias on the mV scale, a hexagonally dense-packed pattern with 2.8 Å periodicity is detected that corresponds to a (1 × 1) structure with respect to Pt(111). At higher bias, a 30° rotated structure with 4.8 Å periodicity and (√3×√3)­R30° symmetry with respect to the support shows up. A DFT-based structural search based on a genetic algorithm identified a single-stack Cr_3_O_6_ trilayer to perfectly reproduce these experimental results (Figure 1d).^20^ The dense (1 × 1) structure hereby reflects the topmost oxygen plane, while the (√3×√3)­R30° pattern is introduced by the central Cr plane that comprises ^1^/_3_Cr^4+^ and ^2^/_3_Cr^3+^ ions. As Cr^4+^ ions have a lower state-density near the Fermi level, they appear with reduced contrast in the STM and produce the observed (√3×√3)­R30° superstructure pattern.

**Figure 1:**
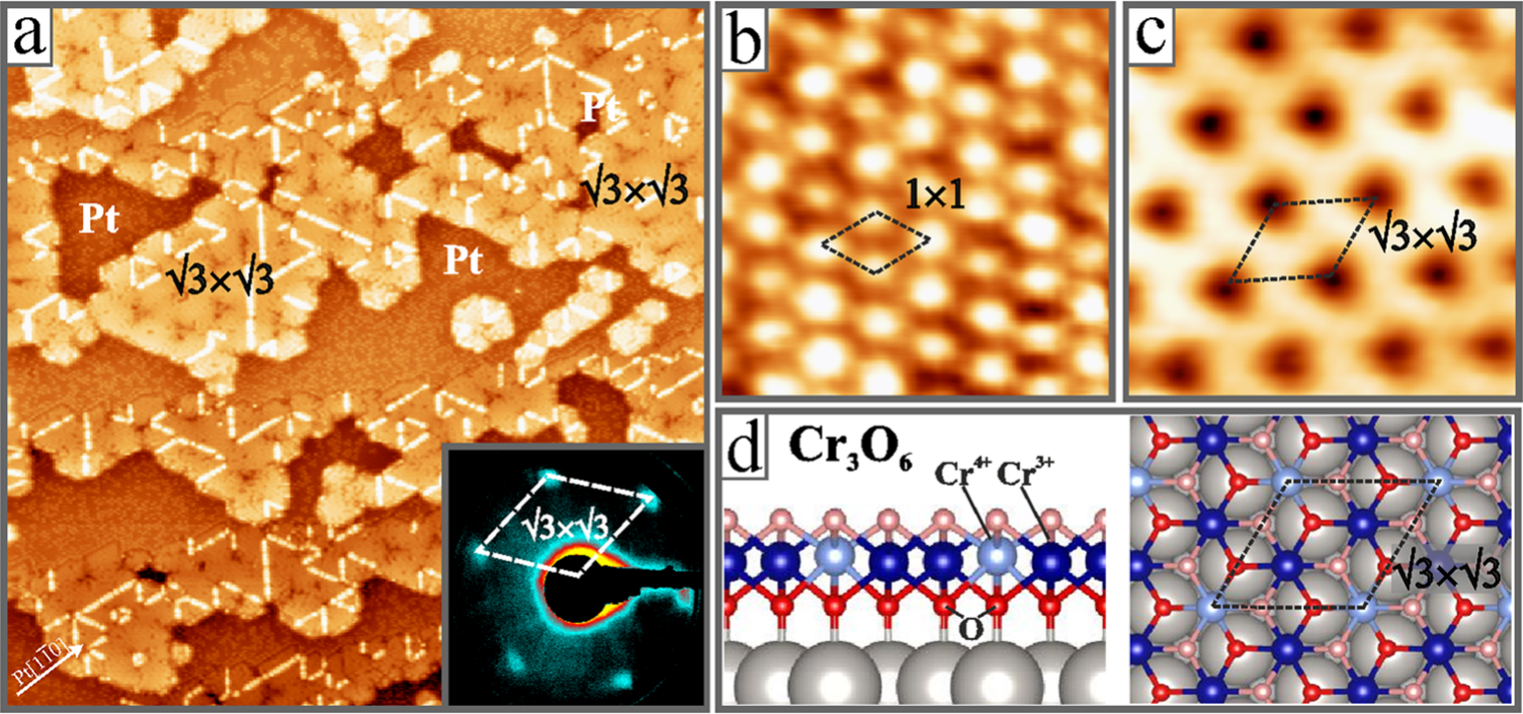
(a) Overview (100 × 100 nm^2^, U_B_ = 0.5 V, I = 0.1 nA) and high-resolution STM images of a Cr_3_O_6_/Pt­(111) film (2.0 × 2.0 nm^2^, U_B_ = 0.1 V) showing (b) the topmost O plane with (1 × 1) symmetry and (c) the interfacial Cr layer with its (√3×√3)­R30° superstructure. The inset in (a) shows a LEED pattern of the film measured at 29 eV. (d) Side- and top-view of the DFT-derived structure of the Cr_3_O_6_ single-stack film on Pt(111).

The (√3×√3)­R30° Cr_3_O_6_ film can be converted to a (2 × 2) configuration by vacuum annealing the sample at 650 K (Figure 2a). In the process, the surface coverage of the film halves while its apparent height increases from 2.8 to 4.5 Å, indicating the transformation from a single-stack to a double-stack geometry. Atomically resolved STM images reveal a Kagome structure at low bias and either a hc- or (2 × 2) pattern at higher bias (Figure 2b-d). The (2 × 2) pattern evolves from the hc-lattice if only every second cation of a six-membered ring contributes to the image contrast. DFT calculations identified the (2 × 2) film as Cr_6_O_11_ double-stack structure, in which the O–Cr–O trilayer discussed before gets capped by a Cr_2_O_3_ hc-plane made of tetrahedrally and octahedrally coordinated Cr ions (Figure 2f).^20^ The alternating Cr coordination induces modulations in the density of states and gives rise to the typical (2 × 2) contrast seen in the STM, while the Kagome lattice is detected for tip configurations that are sensitive to the O anions in the hc-plane (Figure 2b).

**Figure 2:**
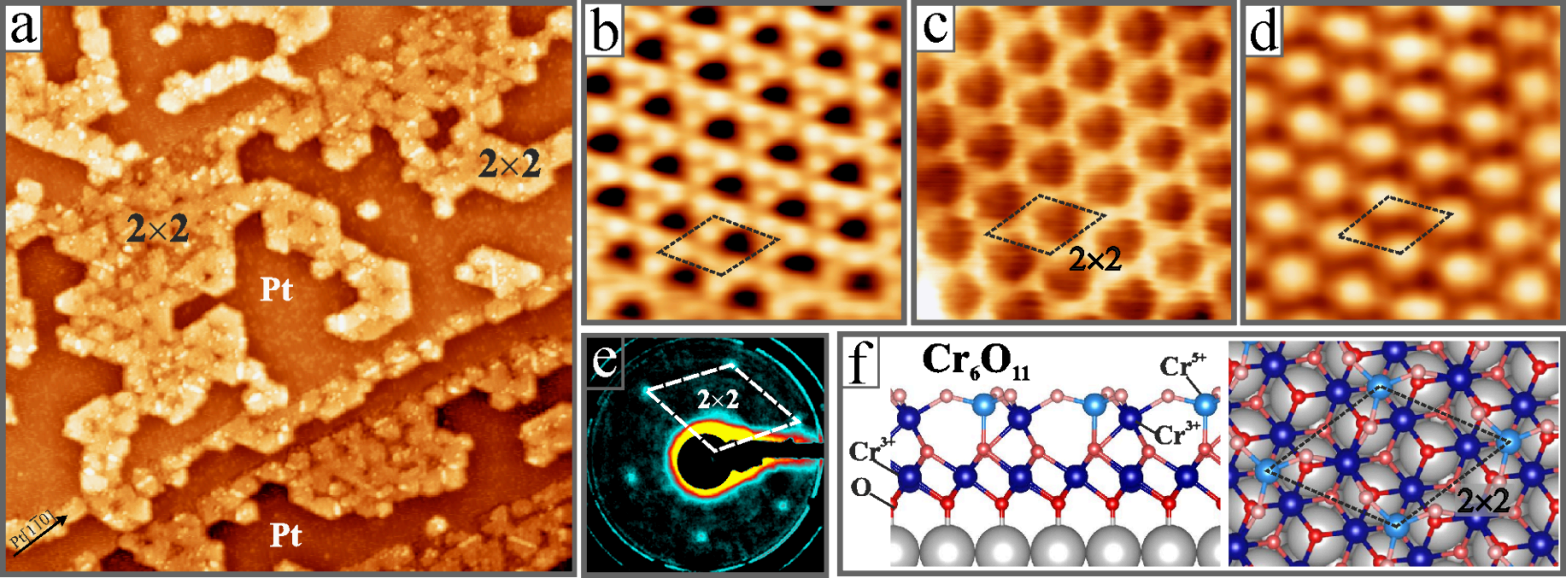
(a) Overview (100 × 100 nm^2^, U_B_ = 0.8 V, I = 0.1 nA) and high-resolution STM images of a Cr_6_O_11_/Pt­(111) film (3.0 × 3.0 nm^2^) showing (b) the oxygen Kagome lattice (U_B_ = 0.05 V), (c) the hc-pattern of the topmost cations (U_B_ = −0.1 V), and (d) the (2 × 2) pattern that appears if only every second cation is imaged (U_B_ = 1.2 V). (e) LEED pattern of the film measured at 29 eV. (f) Side- and top-view of the DFT-derived structure of the Cr_6_O_11_ double-stack film on Pt(111).

To address workfunction changes between (√3×√3)­R30° and (2 × 2) Cr-oxide films and deduce information on the underlying electric dipole, STM field-emission-resonances (FERs) and apparent barrier-height (d­(*lnI*)/d*z*) maps were measured. The former reflect vacuum states that develop in the classical part of the tip–sample junction at bias voltages above the workfunction (Figure 3).^23,24^ The resonance condition is given by the quantization of free-electron wave functions in the triangular potential formed between the downward sloping vacuum energy and the sample surface:

$$E_{n}=\varphi +{\left(\frac{3\pi \hbar e}{2\sqrt{2m}}\right)}^{2/3}F^{2/3}{\left(n-\frac{1}{4}\right)}^{2/3}$$ 1

with *F* the electric field and *n* the quantum number. The first FER experiences an additional downshift due to image-potential interactions in the tip–sample junction. To account for this effect and increase the reliability of fitting, we have added a constant offset of 0.5 eV to the first FER. The procedure to determine this offset is introduced in the Supporting Information (SI). Apparent barrier-height spectroscopy exploits the response of the tunneling current *I* to a modulated tip height d*z*, according to $\varphi =\frac{\hbar^{2}}{8m}{\left(\frac{d\ln I}{dz}\right)}^{2}$ . While the FER data give quantitative insight into the local workfunction,^21^ only relative changes can be retrieved from d­( *lnI* )/d*z* maps because of a simplified description of the tunneling barrier.^22,25^

**Figure 3:**
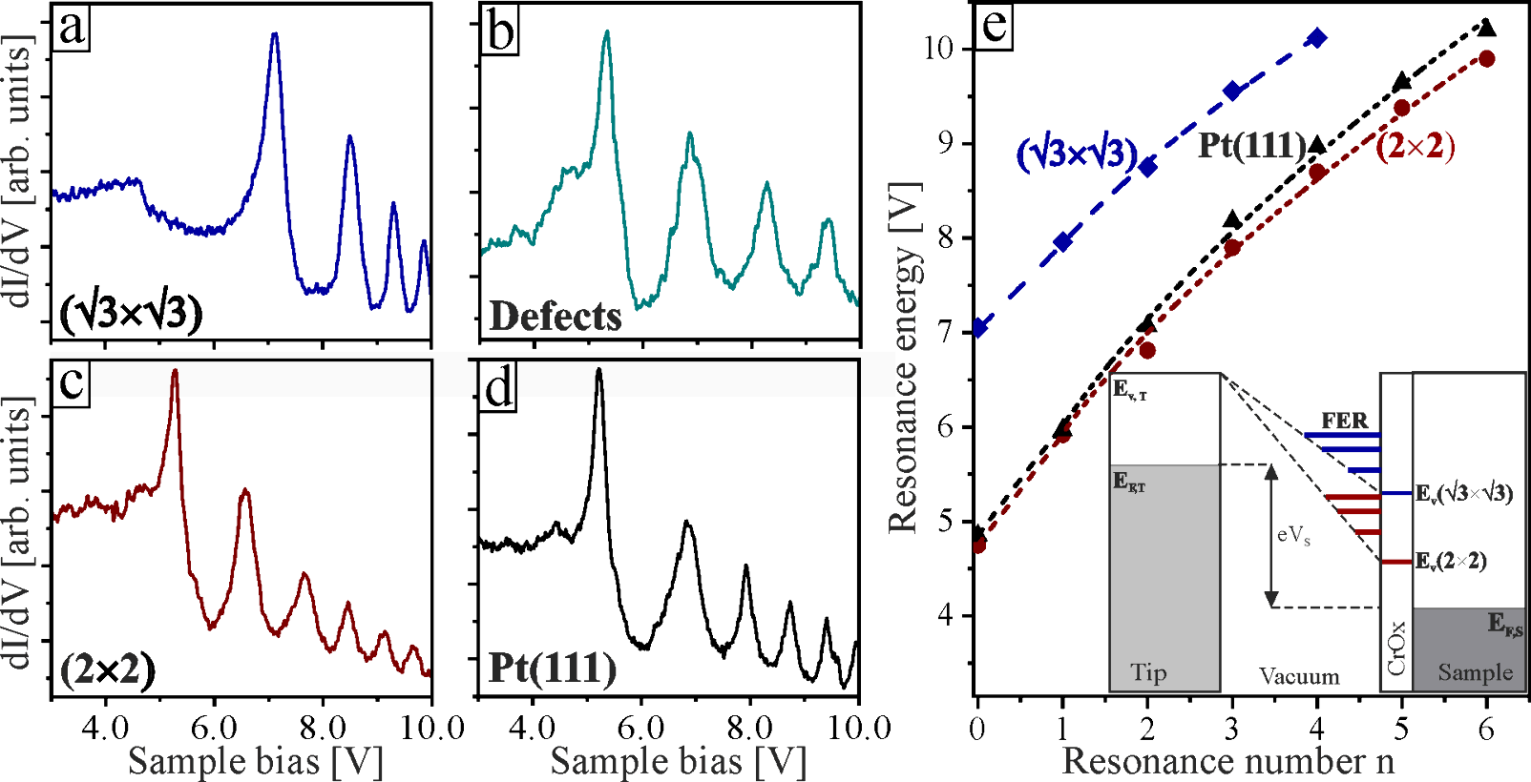
FER spectra measured with 0.1 nA tunneling current and enabled feedback loop on (a) ideal Cr_3_O_6_ single-stacks, (b) Cr_3_O_6_ with embedded line defects, (c) Cr_6_O_11_ double-stacks, and (d) bare Pt(111). (e) Fit of the FER energies to eq 1 and potential diagram of the STM junction, linking FERs to the sample workfunction.

Figure 3 displays several FER spectra taken on single- and double-stack CrO_*x*_/Pt­(111) films, as well as on pristine Pt(111). Spectra taken on single-stack patches typically have their first FER at ∼7 V, with three more states following in the accessible bias range of our STM electronics (Figure 3a). Only above line defects in the (√3×√3)­R30° film, low-lying FERs starting at ∼5.2 V are detected, suggesting a local workfunction decrease induced by the defects (Figure 3b). Conversely, the first FER on bilayer (2 × 2) films appears already at ∼ 5 V and up to five resonances follow at higher bias (Figure 3c). This behavior is comparable to the one observed on bare Pt(111), where the first FER occurs at ∼ 5.1 V (Figure 3d). A quantitative analysis based on eq 1 yields approximate workfunction values of (4.7 ± 0.1) and (4.8 ± 0.1) eV for double-stack (2 × 2) films and bare Pt(111), respectively. In contrast, the (√3×√3)­R30° patches show a substantially higher ϕ value of (7.1 ± 0.2) eV.

Similar conclusions can be drawn from d­( *lnI* )/d*z* maps, as depicted in Figure 4. The topographic image shows a mixed surface, exposing singe- and double-stack Cr-oxide domains together with bare Pt(111). In the barrier-height map, high contrast (bright yellow) is revealed for the (√3×√3)­R30° regions, while minimum intensity is measured for (2 × 2) domains (dark blue). The exposed metal surface takes an intermediate d­( *lnI* )/d*z* value. The deviating electronic nature of single- and double-stack oxide patches becomes evident also in differential conductance (dI/dV) maps of the surface, as displayed in Figure 4b. To maximize the contrast, the maps were taken at 0.8 V sample bias, where the (2 × 2) Cr_6_O_11_ shows enhanced state-density due to unfilled *3d*_*z2*_ levels located on the Cr^5+^ ions of the hc-top plane.^20^ Correspondingly, the (2 × 2) regions are readily discernible by their high dI/dV signal, while (√3×√3) R30° patches and bare Pt(111) leave no clear fingerprint. All measurements presented so far suggest that single- and double-stack CrO_*x*_/Pt­(111)-oxide films have strongly deviating electronic properties, with the former showing a much higher workfunction than the latter.

**Figure 4:**
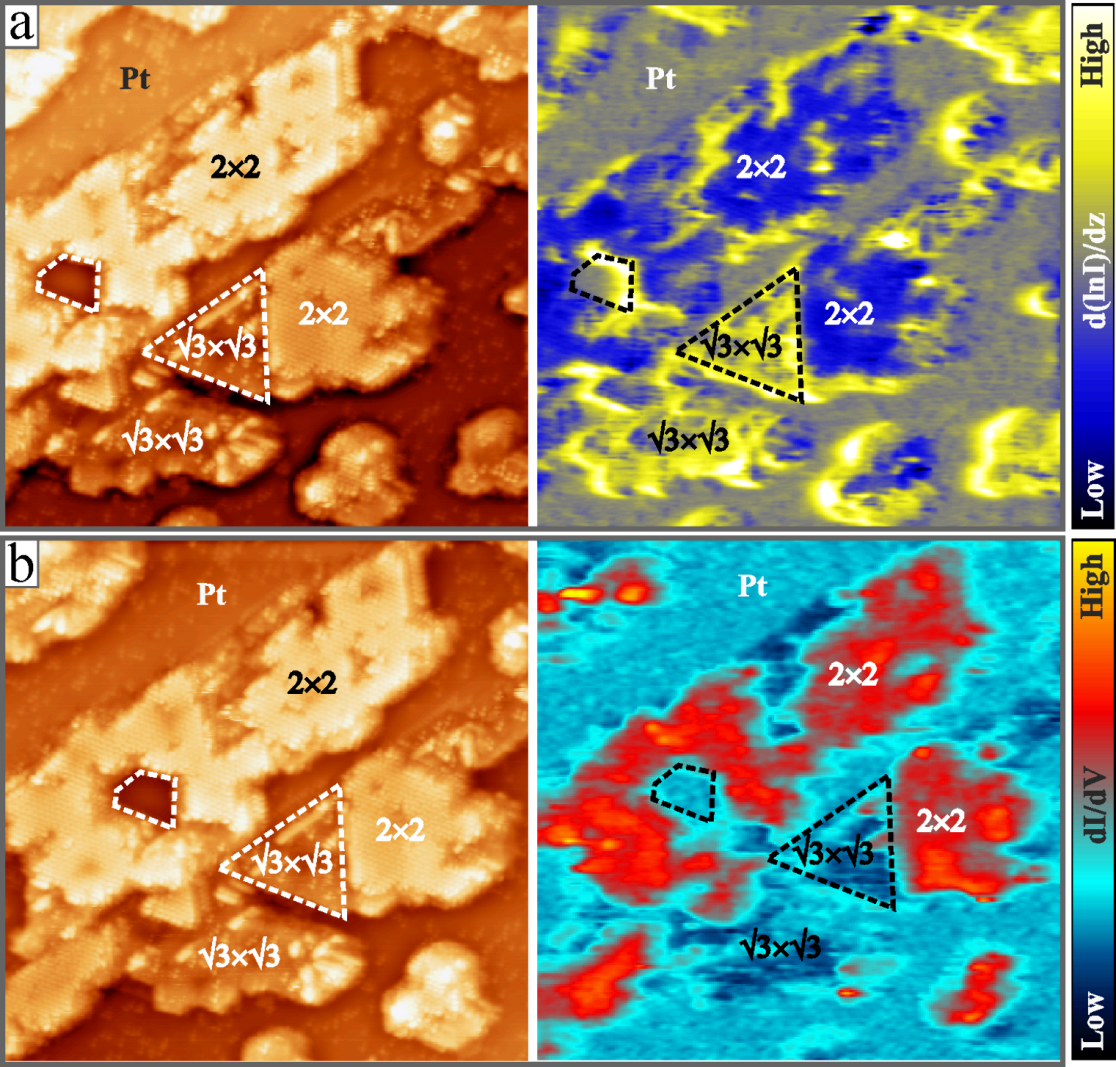
(a) Topography and d­(*lnI*)/dz map of Pt(111) covered with single- and double stack Cr-oxide patches (50 × 50 nm^2^, U_B_ = 0.8 V, I = 0.1 nA). (b) Topography and dI/dV map acquired on the same sample region (50 × 50 nm^2^, U_B_ = 0.8 V, I = 0.1 nA). At the preset parameters, the double-stack Cr_6_O_11_ patches exhibit the lowest workfunction and the highest dI/dV signal. Additional measurements can be found in the SI.

The geometric and electronic properties, calculated for Cr_3_O_6_ single-stack and Cr_6_O_11_ double-stack films with DFT, are compiled in our earlier work and sketched in Figures 1 and 2.^20^ Here, we recall the calculated workfunction of bare Pt(111) (ϕ_Pt_ = 6.1 eV) and the two supported films (ϕ_Cr3O6_ = 8.0 eV and ϕ_Cr6O11_ = 5.7 eV). Apparently, the growth of a Cr_3_O_6_ single-stack film increases the Pt(111) workfunction by +1.9 eV, while the Cr_6_O_11_ double stack leads to a workfunction drop of −0.4 eV. These findings correspond well to our experimental results, yielding workfunction changes of +2.3 eV and −0.1 eV in the presence of single- and double-stack oxide films, respectively. The absolute workfunction values obtained experimentally are ∼1 eV lower than calculated ones, however, a known deficiency of the FER-based approach. The reason behind this discrepancy is the one-dimensional (1D) description of the confinement potential that contrasts to the 3D nature of the actual tip–sample junction.^21,25^ The calculated workfunction of Pt(111), on the other hand, corresponds well to values determined by photoemission and Kelvin probe spectroscopy.^26,27^ We emphasize that relative workfunction changes induced by the different oxide films are well reproduced by FER data and d­( *lnI* )/d*z* maps (Figures 3, 4), and perfectly match the calculated workfunction order, ϕ_Cr3O6_ > ϕ_Pt_ > ϕ_Cr6O11_. Also, the exceptionally high workfunction of single-stack Cr_3_O_6_/Pt films is clearly revealed in both cases, highlighting the strongly electronegative character of this oxide/metal system. In the following, we discuss how the charge-distribution within multistack oxide films governs the development of surface dipoles and the overall film stability.

## TMO_2_/Pt Trilayers

The Cr_3_O_6_ single-stack film features an exceptionally high workfunction compared to other Pt-supported TMO_2_ trilayers along the *3d* transition-metal series (TM = Ti, V, Mn, Fe) (Figure 5a). Many of these structures were actually synthesized on late-transition and noble-metal supports, however, no ϕ measurements were reported.^20,28–30^ The peculiar behavior of the CrO_2_/Pt trilayer leads to a strongly nonmonotonic workfunction trend along the TM series, with ϕ_MnO2/Pt_ being much lower than ϕ_CrO2/Pt_ for example. Figure 5a provides a link between the workfunction and the interfacial charge transfer and helps identifying its two origins. The first one relates to the O–Pt interface bonds, the strengthening and shortening of which progressively increase the positive charge on the Pt substrate. The effect is best seen in TiO_2_/Pt, VO_2_/Pt, and MnO_2_/Pt, where all cations maintain their 4+ oxidation state. The second component, which further enhances the substrate charging for CrO_2_ and FeO_2_ trilayers, involves a reduction of their cations via electron donation from the Pt(111). It is this contribution that is largely responsible for the exceptionally high workfunction of CrO_2_/Pt, where Cr^4+^ and reduced Cr^3+^ cations coexist, as discussed before (Figure 1d).

**Figure 5:**
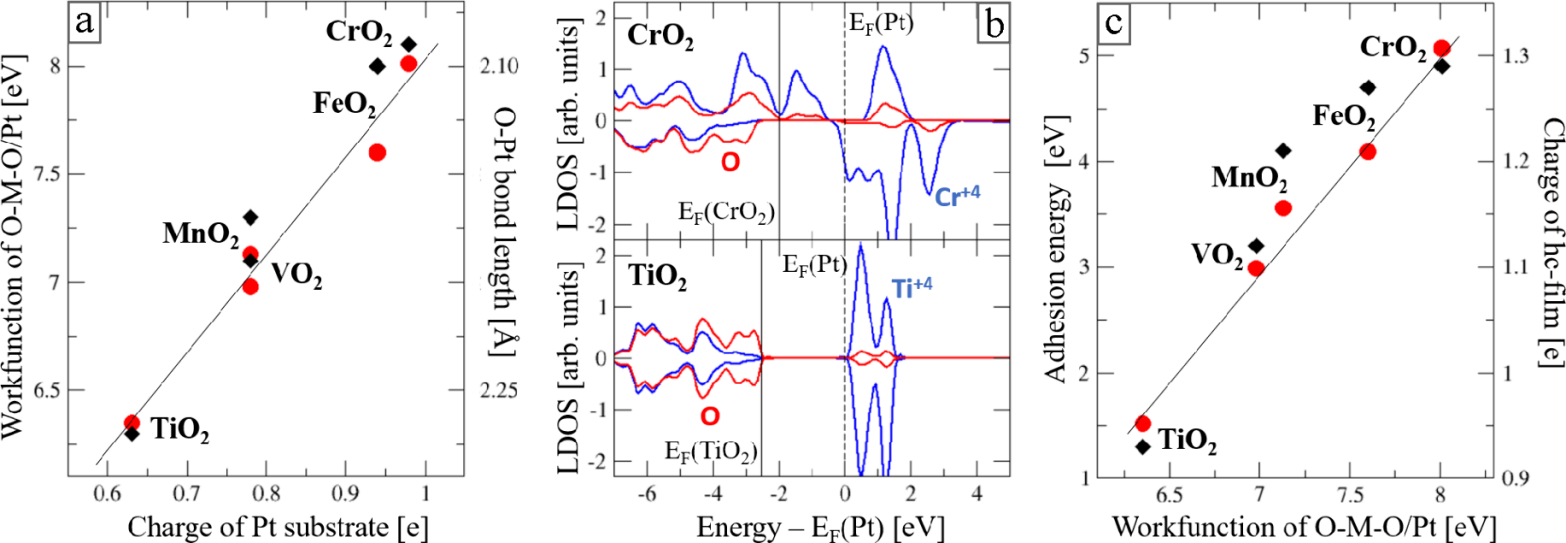
(a) Workfunction of TMO_2_/Pt trilayers (red circles) and interfacial d_O–Pt_ bond length (black diamonds) as a function of the Pt(111) Bader charge (TM = Ti, V, Mn, Cr, Fe). (b) Calculated local density of states (LDOS) of anions (red) and cations (blue) in freestanding CrO_2_ and TiO_2_ trilayers on an energy scale obtained by aligning the common vacuum levels. Fermi-level positions of the oxide films and the Pt(111) substrate are marked by solid and dashed lines, respectively. (c) Adhesion energy (red circles) and Bader charge (black diamonds) of the capping hc-Cr_2_O_3_ film as a function of the workfunction of TMO_2_/Pt trilayers. All lines serve as guides to the eye.

To assess the origin of this specific charge-transfer component, we contrast two markedly different cases: the TiO_2_/Pt system (ϕ_TiO2/Pt_ ∼ 6.4 eV) where all cations maintain their 4+ oxidation state, and the CrO_2_/Pt one (ϕ_CrO2/Pt_ ∼ 8.0 eV), where Cr^3+^ and Cr^4+^ ions coexist. Both trilayers exhibit a similarly high workfunction of 8.1–8.5 eV in freestanding geometry, in which a positively charged cation plane in 4+ oxidation state is sandwiched between two negatively charged oxygen planes. They consequently generate comparably large band offsets (∼2.5 eV) with respect to Pt(111), suggesting similar interface charging effects. However, while empty Cr states are available below the Pt Fermi level, enabling charge transfer and Cr reduction, the TiO_2_ trilayer lacks such empty states and electron transfer is prevented (Figure 5b). It is therefore the low conduction-band edge and the small bandgap of the CrO_2_ trilayer that is responsible for the remarkably high workfunction of the CrO_2_/Pt system.

## Cr_6_O_11_ Double-Stacks

The high stability of Cr_6_O_11_/Pt double-stacks in a wide range of oxygen conditions, as discussed in ref 20., directly reflects the tendency of the strongly electronegative CrO_2_/Pt trilayer to be capped by a Cr_2_O_3_ hc-bilayer. While additional Cr–O bonds between the hc-plane and the trilayer contribute to this stability, it is again the electron transfer that plays the key role. This is best seen when comparing the properties of different TMO_2_/Pt trilayers (TM = Ti, V, Mn, Cr, Fe) after capping them with identical Cr_2_O_3_ hc-planes (Figure 5c). The computational results demonstrate a clear correlation between the Cr_2_O_3_ adhesion (*E*_*adh*_), its charge state (*q*_*s*_), and the workfunction of the TMO_2_/Pt support. The CrO_2_/Pt system with its unusually high workfunction of 8.0 eV clearly features the largest *E*_*adh*_ and *q*_*s*_ values. In contrast, the TiO_2_/Pt system, characterized by the lowest workfunction (ϕ_TiO2/Pt_ = 6.4 eV), also exhibits the lowest *q*_*s*_ and *E*_*adh*_ values. The large band offset between the CrO_2_/Pt trilayer (ϕ_CrO2/Pt_ = 8.0 eV) and the freestanding hc-plane (ϕ_Cr2O3_ = 5.7 eV) triggers an electron transfer from one of the hc−Cr ions, that switches to a 5+ charge state, to the underlying trilayer, resulting in a substantial stabilization of the Cr_6_O_11_ double-stack. Our findings indicate that the band-offset argument, previously used to rationalize the electron distribution at various metal-oxide interfaces,^31^ also applies to these more complex systems.

In conclusion, by means of STM spectroscopy and DFT calculations we have demonstrated that single-stack Cr_3_O_6_ films on Pt(111) exhibit a much higher workfunction than Cr_6_O_11_ double-stacks. The exceptionally high ϕ value of the former arises from a sizable electron donation from the Pt substrate, which triggers a partial reduction of Cr ions in the trilayer from 4+ to 3+, produces a strong negative surface dipole and confers a strongly electronegative character to this support. Capping the trilayer with a Cr_2_O_3_ hc-plane drastically reduces the workfunction, as the hc-layer becomes the main electron source for the charge-accepting CrO_2_/Pt stack and the orientation of the surface dipole is reversed. The interlayer charge exchange is also responsible for the enhanced stability of the double-stack oxide film. The band-offset argument suggests similar behavior for all TMO_2_ trilayers that exhibit empty states below the Fermi level of the metal support and behave as electron acceptors. Further insight into the charge-transfer characteristics of oxide bilayer films may be obtained from Kelvin probe techniques that provide better resolved workfunction data than the STM-based methods employed here.^32^

## Experimental and Theoretical Methods

The experiments have been performed in an ultrahigh vacuum chamber equipped with a Pan-type STM operated at 100 K and standard tools for sample preparation and thin-film deposition. A Pt(111) single crystal, Ar^+^ sputtered at 1500 eV and annealed at 1000 K, was used as substrate for the oxide growth. The (√3×√3)­R30° Cr_3_O_6_ oxide film was prepared by vacuum deposition of 1 ML Cr (99.9% purity) and annealing at 650 K in 1 × 10^–6^ mbar O_2_ via chamber backfilling. The (2 × 2) Cr_6_O_11_ phase was formed by vacuum-annealing the Cr_3_O_6_ at 650 K.^20^ Both phases displayed sharp electron-diffraction patterns, the first one with (√3×√3)­R30° and the second with (2 × 2) symmetry. STM images were acquired in the constant current mode, using electrochemically etched gold tips. The local work-function was probed with two lock-in amplifier-based approaches: (i) measuring FERs in the tip–sample junction with enabled feed-back loop (ΔV = 20 mV), and (ii) acquiring apparent barrier height maps from the logarithmic current response to a modulated tip height (Δz = 0.5 Å). While the first scheme delivers absolute ϕ values, only relative trends are accessible with the second method.

The DFT calculations were performed with the Vienna Ab-initio Simulation Package,^33,34^ using the Projector Augmented Wave method to represent electron–core interactions and a 400 eV energy cutoff for expanding the Kohn–Sham orbitals.^35,36^ A dispersion-corrected exchange-correlation functional (optB88 vdW)^37,38^ was employed within the DFT+*U* approach proposed by Dudarev.^39^ The *U* values were set similar to our previous studies.^20,40^ All calculations were spin-polarized and the relative stability of nonmagnetic versus magnetic solutions, either with parallel or antiparallel spin moments, was systematically tested. Ionic charges were estimated with the Bader partition scheme,^41,42^ and magnetic moments were obtained by integrating the spin density within the Bader volumes. The Pt(111) substrate was represented by four atomic planes (2.77 Å in-plane lattice constant) with the oxide film placed at one side of the slab. The periodic images were separated by 10 Å of vacuum and dipole corrections were applied. The Brillouin zones of (√3×√3)­R30° and (2 × 2) cells were sampled on (7 × 7 × 1) and (6 × 6 × 1) Monkhorst–Pack k-point grids, respectively.

## Supplementary Material





## Notes

Details of the fitting routine used for workfunction determination via FERs and additional workfunction and conductance maps of the CrO_*x*_/Pt­(111) films (PDF)
Transparent Peer Review report available (PDF)
