# Supplementary material for: Unraveling the Inner Electronic Structure of Chromium-Oxide Films by Probing the Layer-by-Layer Evolution of Their Workfunction
Source: J Phys Chem Lett. 2025 Jul 7;16(28):7222–8. doi: 10.1021/acs.jpclett.5c01617 (PMC12278308; doi:10.1021/acs.jpclett.5c01617)
Supplement: Supplementary file 1 [file jz5c01617_si_001.pdf]

Supporting Information

Unraveling the Inner Electronic Structure of Chromium-Oxide Films by Probing the Layer-by-Layer Evolution of their Workfunction

Ghada Missaoui,[a] Jacek Goniakowski,[b,] Claudine Noguera,[b] Niklas Nilius [a,*]*

[a] *Carl von Ossietzky Universität Oldenburg, Institut für Physik, D-26111 Oldenburg, Germany*
[b] *CNRS-Sorbonne Université, Institut des Nanosciences de Paris, UMR 7588, F-75005 Paris, France*
Corresponding authors: jacek.goniakowski@insp.jussieu.fr, niklas.nilius@uol.de

1. FER-Fitting for Workfunction Determination

Field emission resonances (FERs) are the eigenstates E_n of quasi-free electrons localized in the classical part of the tip-sample junction of an STM. At glance, the confining potential can be considered to be of triangular shape, delimited by the sample surface and the vacuum energy that slopes down with the tip-electric field. The corresponding resonance condition reads: $E_n = \varphi + \left(\frac{3\pi\hbar e}{2\sqrt{2m}}\right)^{2/3} F^{2/3}\left(n-\frac{1}{4}\right)^{2/3}$ (Eq. 1), whereby ϕ is the bottom of potential and defines the sample workfunction, F is the electric field in the junction, and *(n-1/4)* is the quantum number corrected for the boundary conditions of the potential.[1,2,3,4] While this approach is well justified for higher FER states, larger deviations emerge for the 1st resonance, whose probability density is closest to the surface and whose energy is strongly affected by image-potential interactions. More precisely, electrons in the lowest FER are subject to an attractive coupling to positive image charges induced in the sample surface, here the Pt(111) support. This leads to a sizable downshift of the resonance energy of the order of $\Delta E = -c/4z$, with z being the separation between the FER-electron and its image charge and c a constant. However, neither z nor c can be determined precisely, as the spatial localization of the 1st FER and the screening ability of the oxide layer on top of Pt(111) are unknown.

There are two approaches to overcome this deficiency: (i) ignore the 1st FER that is most affected by image charge effects and conduct the fitting only with higher FERs, or (ii) artificially upshift the 1st FER to compensate for the attractive interactions. We have tested both approaches for data acquired on bare Pt(111), as summarized in Fig. S1 and the associated table S1. Approach (i) gives an intermediated workfunction value of $\phi \sim 4.6$ eV but naturally needs to cope with less data points for fitting. For high workfunction samples, e.g. the (√3×√3)R30° film, only three FER states are available and the resulting fitting errors are relatively large (Fig. S1, panel a). Approach (ii) enables a more stable fitting procedure, however, realistic offsets of the 1st FER to compensate image-potential effects need to be determined prior to fitting. In our work, we have systematically tested the impact of level upshifts on the determined workfunction, using again the data acquired on Pt(111) (Fig. S1, panel b-f). The smallest fitting error for ϕ is obtained for 0.5 eV offset on the 1st FER (Table S1), which also gives the smoothest behavior upon visual inspection of the fitting (panel d). We therefore used a constant offset of 0.5 eV to fit all FER spectra in our work, including the ones obtained on the different oxide films.

Note that the Pt(111) workfunction fitted with the above constraints ($\phi \sim 4.75$ eV) is about 1 eV lower than macroscopic values measured with photoemission spectroscopy or Kelvin probe techniques.[5,6] Main reason

for this discrepancy is the use of a simplified one-dimensional potential to fit the FERs, which clearly deviates from the three-dimensional geometry of a real tip-sample junction.

Table S1: Workfunction ϕ, electric field values F and corresponding error margins obtained from fitting Pt(111) field-emission resonances to different modifications of equation 1.

**Equation 1 with**	**Workfunction ϕ in eV**	**Electric field F in V/m**
1st FER neglected	4.60±0.20	2.59×10^9
1st FER upshifted by 0.0 eV	4.30±0.24	2.83×10^9
1st FER upshifted by 0.25 eV	4.52±0.17	2.65×10^9
1st FER upshifted by 0.5 eV	4.78±0.11	2.48×10^9
1st FER upshifted by 0.75 eV	4.95±0.12	2.31×10^9
1st FER upshifted by 1.0 eV	5.17±0.18	2.14×10^9

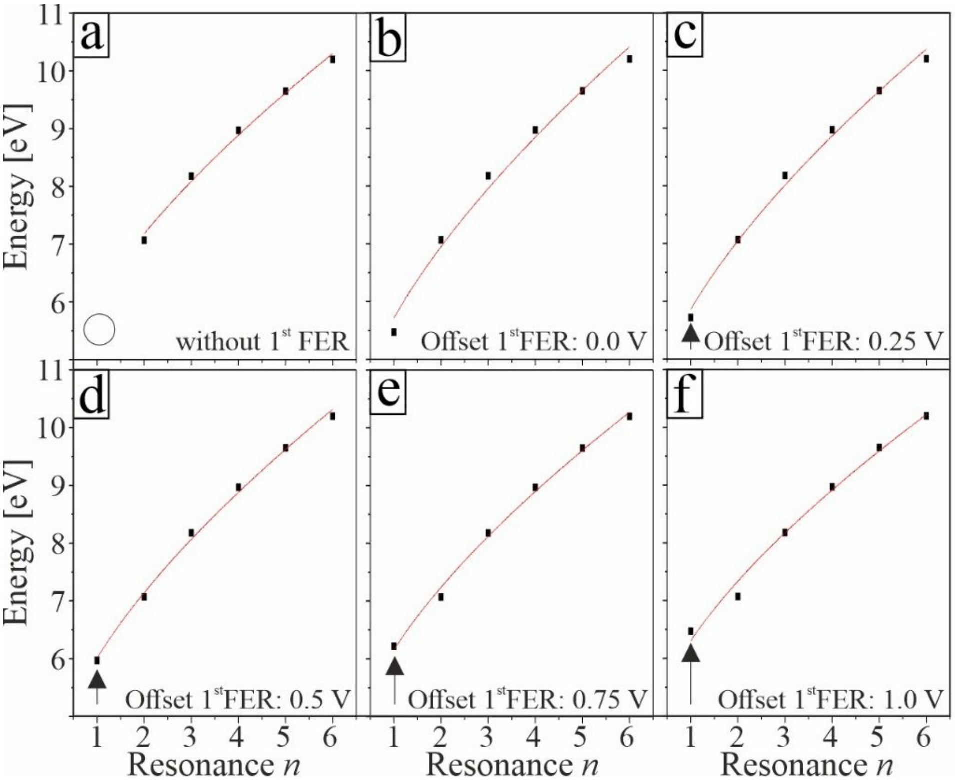

Figure S1: Fitting of FERs measured on top of bare Pt(111) at different conditions: (a) disregarding the 1st FER, (b-d) off-setting the 1st FER by values between 0.0 and 1.0 eV.

Using the fitting procedure developed above, we have determined workfunction values for Pt(111), the single-stack and double-stack oxide film (Table S2). As discussed in the main text, the Cr_3O_6 single-stack film has an exceptionally high workfunction. In contrast, the Cr_6O_{11} double-stack gives rise to the smallest electric field in the tip-sample junction upon probing the FERs. The latter finding corresponds to the larger topographic height of the double-stack with respect to the single-stack film.

Table S2: Workfunction ϕ and electric fields F obtained from FER data measured on Pt(111), Cr_3O_6/Pt(111) and Cr_6O_{11}/Pt(111) samples. The values were obtained from Eq. 1, by upshifting the 1st FER by 0.5 eV.

Sample	Workfunction ϕ in eV	Electric field F in V/m
Pt(111)	4.78±0.11	2.48×10^9
Cr_3O_6/Pt(111)	7.11±0.21	2.25×10^9
Cr_6O_{11}/Pt(111)	4.72±0.14	$1.5\text{x}10^9$

2. Additional experimental data

To corroborate our conclusions on the specific workfunction characteristic of single-stack and double-stack CrO_x films on Pt(111), we display another data set here. Similar to Fig. 4 in the main text, the (√3×√3)R30° regions (single-stack Cr_3O_6) exhibit the largest signal in the *d(lnI)/dz* map, indicating the highest workfunction (Fig. S2a). Conversely, the (2×2) patches (double-stack Cr_6O_{11}) stick out by their large dI/dV signal in conductance maps, being related to the high empty state-density induced by the Cr^{5+} ions in the honeycomb top layer (Fig. S2b).

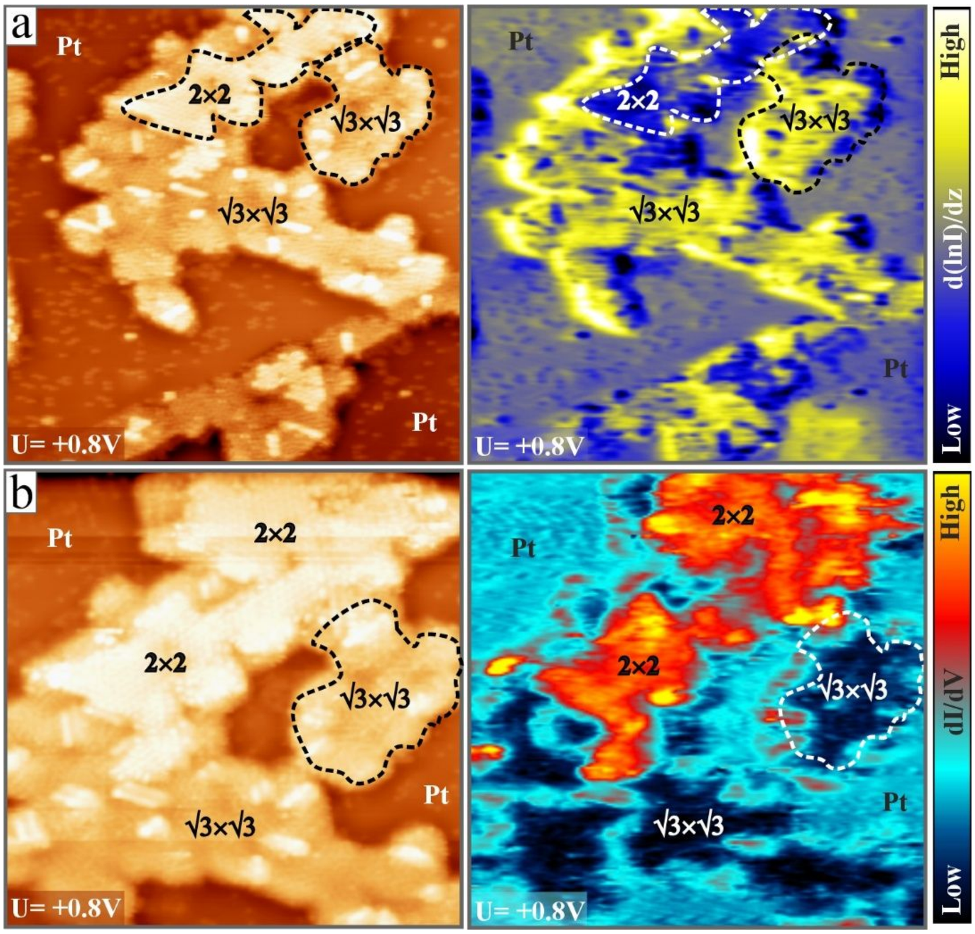

Figure S2: **(a)** Topographic and *d(lnI)/dz* map of Pt(111) covered with single- and double stack Cr-oxide patches (40×40 nm^2, U_B = 0.8 V, I = 0.1 nA). **(b)** Topographic and dI/dV map acquired on the same sample region (30×30 nm^2, U_B = 0.8 V, I = 0.1 nA).

List of References

(1) Gundlach, K. H. Zur Berechnung des Tunnelstroms durch eine trapezförmige Potentialstufe. *Sol. Stat. Electron*. **1966**, *9*, 949-957.

(2) Rienks, E. D. L.; Nilius, N.; Rust, H. P.; Freund, H.-J. Surface potential of a polar oxide film: FeO on Pt(111). *Phys. Rev. B* **2005**, *71,* 241404.

(3) Huang, Z.; Zhen X.; Junyi Z.; Haoran C.; Wenhui R.; Yuxuan L.; Xiaojie W.; Hao Z.; Kai W. Local work function measurements of thin oxide films on metal substrates. *J. Phys. Chem. C* **2019,** *123,* 17823–17828.

(4) Aladyshkin, A. Y. Quantum-well and modified image-potential states in thin Pb(111) films. *J. Phys.: Condens. Matter* **2020**, *32*, 435001.

(5) Kawano, H. Effective work functions of the elements. *Prog. Surf. Sci.* **2022**, *97*, 100583.

(6) Derry, G.N.; Kern M. E.; Worth, E. H. Recommended values of clean metal surface work functions. *J. Vac. Sci. Technol. A* **2015**, *33*, 060801.
