# Supplementary material for: Unraveling the Inner Electronic Structure of Chromium-Oxide Films by Probing the Layer-by-Layer Evolution of Their Workfunction
Source: J Phys Chem Lett. 2025 Jul 7;16(28):7222–8. doi: 10.1021/acs.jpclett.5c01617 (PMC12278308; doi:10.1021/acs.jpclett.5c01617)
Supplement: Supplementary file 2 [file jz5c01617_si_002.pdf]

jz-2025-01617c.R1

Name: Peer Review Information for "Unraveling the Inner Electronic Structure of Chromium-Oxide Films by Probing the Layer-by-Layer Evolution of their Workfunction"

First Round of Reviewer Comments

Reviewer: 1

Comments to the Author

The manuscript by Missaoui et al. presents LWF measurements of CrOx films on Pt(111) using two STS-related methods, complemented by mechanistic insights by DFT calculations. The study is well-structured, with comprehensive experimental and theoretical data that are rigorously analyzed. I recommend acceptance in JPCL after addressing the following minor revisions:

1) As the structural details of Cr2O3 and Cr6O11 films have been previously reported in Ref. 21, the overview images in Figures 1 and 2 may be redundant. Additionally,

LEED pattern is not inset in (a) as mentioned in Figure 2

2) Given that FER spectra are highly sensitive to tip conditions and tunneling parameters, which are crucial for the LWF measurements, the authors should provide more detailed experimental parameters and conditions.

3) Figures 4a and 4b appear to contain identical images, which should be corrected or clarified.

Reviewer: 2

Comments to the Author

The manuscript written by Missaoui et. al. deals with the work function (WF) of atomically flat Cr-oxide single- (Cr3O6) and double-stack films (Cr6O11) on the Pt(111) surface. The WF was measured by UHV STM spectroscopy, while variations of the WF were imaged by effective barrier-height (d(lnI)/dz) mapping. All measurements were done at 100 K. Assisting DFT calculations were used to understand basic mechanisms involved in WF phenomena. – To summarize, surface regions with Cr3O6/Pt(111) exhibit a very high WF of about 7 (exp.) or 8 eV (theo.) (!), while surface regions of Cr6O11/Pt(111) have the same WF as the uncovered Pt(111) surface (5.7 eV, exp. and theo.). The very high WF of Cr6O11/Pt(111) is due to an electron transfer from the Pt substrate into the film, which leads to a partial reduction of the Cr ions from 4+ to 3+ and thus creates a strong negative surface dipole. When the second stack is placed on top of the first, the latter charge transfer is eliminated as this top layer releases the charge. As a result, the dipole is reversed and phi_Cr6O11/Pt(111) is smaller than phi_Cr3O6/Pt(111). After the comparison between experiment and theory, theory is further used to compare this oxide film with other TMO2 trilayer films such as TiO2, VO2, MnO2 and the like.

Metal-supported thin oxide films are a prerequisite for fundamental research into oxide materials and associated applications in heterogeneous catalysis. For a comprehensive understanding of oxide films and associated surface processes that take place on such thin films, every type of film property must be known. In particular, the WF plays an important role as it determines, for example, the adsorption or growth of metal nanoparticles (NPs) on the films as well as the electronic properties of the NPs such as the charge state. The work presented here makes a very important contribution to the study of Cr-oxide films, as WF measurements on metal-supported oxide films are quite rare, especially when performed with STM. The quality of the data presented here is high and I would like to take this opportunity to congratulate the authors on the experimental results and the comparison with theory. The interpretation of the results is straightforward and convincing and the manuscript is well written and structured. For all these reasons, I recommend publication of the manuscript in J. Phys. Chem. Lett.

However, the manuscript should not be published in its current form; it needs a major revision to clarify some things and **to complete the manuscript**. Here are my critics, comments and questions:

** Main critics, comments and questions **

1.

a) A large ‘strangeness’ of this STM field-emission-resonance (FER) spectroscopy method is this first peak, which has to be lowered by a ‘magic value’, here by 0.5 eV (page 4). In terms of WF, it already is a large value. The authors write ‘~0.5 eV’, so there seems to be an error. - I think that the authors have to justify this 0.5 eV value and to describe this phenomena of image-potential effects a bit more precisely. Is there a work that precisely describes this downshift? If so, please, cite this work. What is the error of the value and how does this influence the fits in Fig. 3e?

Note that in Fig. 3e, there are only 3 values after the first one concerning the sqrt(3) x sqrt(3) structure! The fitting therefore strongly depends on the first value and I guess that there easily is an error of +/- 1 eV for the WF of phi_Cr3O6/Pt(111) = 7 eV.

b) In view of a), please, mention an error for all the three exp. WF values (phi_Cr3O6/Pt(111), phi_Cr6O11/Pt(111), phi_Pt(111)).

c) Is the electric field, F, constant at all n’s? Please, justify.

2.

On page 4 it is suggested that effective barrier-height mapping yields a signal (d(lnI)/dz), which is somewhat proportional to sqrt(phi) on the surface. On the top of page 5 it is written that “… an approximate workfunction of 5.6 eV **for both**, the double-stack (2×2) film and bare Pt(111) (from Eq.1)” is found. In this case, I expect no contrast difference in d(lnI)/dz images between regions with the double-stack (2×2) film/Pt(111) and bare Pt(111) surface regions because those regions seem to have the same WF. However, this is not the case, see Fig. 4a (right): there is a clear contrast difference between the Pt(111) (bright) and Cr6O11/Pt(111) regions (dark). Why is this, please clarify.

3.

The term “workfunction” (WF) is not really well defined in this work, in particular not in the experimental section. What do the authors actually measure in the experiment (and calculate in the theory)? I guess that the WF is given by the film **and** the underlying support and not the film alone. However, in many places the reader gets the impression

that only the film is meant, which would be strange. The entire manuscript has to be revised that potential misunderstandings do not appear. Suggestion: just define and clarify the WF somewhere at the beginning of the manuscript by also introducing phi_Cr3O6/Pt(111), phi_Cr6O11/Pt(111) and phi_Pt(111).

4.

Because phi_Cr3O6/Pt(111) is from the film and the Pt(111) support and because Pt(111) already has a very high WF (5.9 eV !) with respect to many other (111) surfaces, even compared to Pd(111), I have the feeling that the high value of phi_Cr3O6/Pt(111) is somewhat also due to the WF of the Pt(111) surface. If one places, in a 'Gedankenexperiment', the same film onto, e.g., Ag(111), the WF should be way much smaller because the WF of Ag(111) is much smaller (4.53 eV!). Is this the case and can this be stated like this?

5.

The authors mention a WF value of phi_Pt(111) = 5.7 eV for the Pt(111) surface on page 6 (line 25). a) I guess that this is the author's calculated value. Is this correct?

b) To my opinion WF values should be always compared with literature values. So, how does the exp. and theo. value of 5.7 eV compare with literature values? The authors could use references [1] and [2]:

[1] Derry, G. N. and Kern, M. E. and Worth, E. H., Recommended values of clean metal surface work functions. J. Vac. Sci. Technol. A 33, 060801 (2015).

[2] Kawano, H., Effective Work Functions of the Elements. Appl. Surf. Sci. 97, 100583 (2022).

c) Are there any WF values previously measured on the Cr-oxide films ? How do compare those values with the ones obtained here?

6.

Page 8: many details are clearly missing in this experimental method section. I consider it incomplete. If more space is needed the authors could use a supporting information document. Please, consider:

a) For all equipment and materials, more information should be supplied. This concerns in particular:

- The STM (model, company, country or self-made with reference)

- The evaporator (e-beam or Knudsen?, model, company, country)

- The Pt(111) crystal (purity, polishing/orientation quality, company, country)

- The Cr material for evaporation (purity, company, country)

- The gases used (content/purity, company, country)

b) Was oxygen backfilled into the UHV chamber or do the authors use a capillary doser in front of the sample?

c) Ar sputtering: which energy did the authors use and how long did the authors sputter?

c) Some more details about STM:

- What was the modulation in z for d(lnI)/dz imaging?

- Is the signal d(lnI)/dz shown in Fig. 4a or sqrt(d(lnI)/dz)?

- dI/dV data: how strong was here the modulation voltage?

** Other comments **

7.

The authors should mention in the abstract and introduction that STM (spectroscopy) was done at 100 K.

8.

Page 2 with “The underlying mechanisms are often inaccessible to direct measurements and can be retrieved only by theoretical means.” Please, check the meaning of the sentence.

9.

Page 2 with “In fact, most experimental techniques are sensitive to global quantities only, such as the mean workfunction that arises from the electron distribution across all layers of the oxide stack.” To my personal opinion, this sounds a bit too negative. There were in the past quite a few SPM works that could reveal and explain WF phenomena at the nm scale. Think about all the Kelvin probe force microscopy (KPFM) work done on oxide thin films. With respect to STM, there is much less, I agree. – In any case it would be interesting to see whether or not KPFM gives the same results as STS.

8.

Figure 2: Why is the quality of the LEED image so low? Do the authors have another image with a higher quality?

9.

Page 6, line 26: “(phiCr3O6 = 8.0 eV and phiCr6O11 = 5.7 eV for)” <= What is coming after ‘for …’ ?

10.

Page 7, line 48: “as discussed Ref. [21]” => “as discussed **in** Ref. [21]”

Reviewer: 3

Comments to the Author

The major advance is the discovery and mechanistic explanation of how the growth of Cr-oxide film on Pt(111) changes the work function through interfacial and interlayer charge transfer. Specifically:

Cr_3O_6 trilayers exhibit an extremely high work function (~7.0 eV) due to a massive electron transfer from the Pt substrate, which partially reduces Cr^{4+} to Cr^{3+} and creates a strong negative surface dipole.

Capping the trilayer with a Cr–O honeycomb layer (forming Cr_6O_{11} double-stacks) reverses the dipole orientation and reduces the work function (~5.6 eV) by diminishing electron transfer from Pt, stabilizing the structure.

This behavior is generalizable to other transition metal oxides, showing that interfacial and interlayer charge redistribution governs both the work function and stability of oxide/metal systems.

The immediate significance is that it:

Demonstrates tunable work function engineering by changing oxide stacking sequences, offering a pathway for customizing surface electronic properties.

Links charge transfer mechanisms to structural stability, a critical insight for designing robust, functional oxide/metal interfaces.

Provides a predictive framework for other TMO systems where similar interfacial charge dynamics can be exploited to tailor electronic or catalytic behavior.

Is relevant for applications in catalysis, electronic devices, and energy materials, where control of surface potential and interface stability is essential.

Overall this is a great paper, there are just some errors that need corrected including a formula that the authors use incorrectly, see below:

Major and minor changes:

- Equation 1 appears incomplete. It should be written as (n−1/4)2/3. Note that for n≫1, (n−1/4)2/3≈n2/3. The extrapolation of the linear fitting function of Un versus n2/3 for higher-order FER states toward n=0 should provide a reasonable estimate of the work function. For clarification, please refer to Equation 2 in this reference. DOI 10.1088/1361-648X/aba3f0
- For Fig. 1b, Fig. 1c, Fig. 2b, Fig. 2c, and Fig. 2d, please include scale bars on the STM images or clearly indicate the size of each image.

- On page 4, the text states, "The 1st FER is typically downshifted by ~0.5 eV due to image-potential effects in the tip-sample gap." Please provide a reference to support this claim if it is meant as a general observation for all FER measurements.

- In the apparent barrier height map spectroscopy, the modulation amplitude is not specified. Please indicate either the applied AC voltage or the amplitude of tip displacement used in the measurement.

- In Fig. 3e, five data points are shown for the (2×2) system. However, Fig. 3c displays six FER peaks. Please clarify why one data point appears to be omitted.

- In Fig. 3e, no data points are reported for the FERs corresponding to defect (as shown in part b). Please address this omission or include that as well.

- In Fig. 3e, the reported resonance energies (y-axis) do not align with the FER peak positions in the corresponding dI/dV spectra. For example, for the (2×2) and Pt(111) systems, Fig. 3e shows the first FER at ~6.8 V, whereas in the dI/dV spectra, the first FER peaks appear around ~5 V. Please explain or correct this discrepancy.

- In Fig. 3e, the x-axis should be n2/3. As it was mentioned in the second bullet point, extrapolation of the linear fitting function for the dependence Un on n2/3 for the higher-order FER states should be taken into account. Please check Fig. 6 in this paper for reference.

- On page 6, the text states that based on the apparent barrier height map, WF Cr3O6> WF Pt ~ WFCr6O11. If this is accurate, why isn't a similar or comparable contrast observed between Pt and Cr_6O_{11} in the d(ln I)/dz map in Fig. 4? That is, why is there a visible contrast between Pt and Cr_6O_{11} in the ABH map? Please also include a color scale bar next to the map for clarity.

- For Fig. 5b, if the plot shows the projected (calculated) density of states, it would be clearer to label the y-axis as PDOS rather than LDOS, or to mention in the caption that this represents the calculated density of states.

Author's Response to Peer Review Comments:

We thank all three reviewers for their time and efforts to read and evaluate our manuscript and for the large number of insightful and constructive suggestions on how to improve the quality and readability of our work. Please find detailed answers to all your questions in the following list.

Reviewer 1:

Q1: As the structural details of Cr2O3 and Cr6O11 films have been previously reported in Ref. 21, the overview images in Figures 1 and 2 may be redundant. Additionally, LEED pattern is not inset in (a) as mentioned in Figure 2

A1: We would prefer to keep the STM images as an integral part of Figs. 1 and 2, for the following two reasons. (i) It appears important that the reader gets a quick overview on the nature and quality of the Cr-oxide films discussed in this work, without the need to check our earlier reference No. 21. (ii) As we are working on this oxide system for more than a year now, the quality of the STM measurements has improved substantially with respect to our previous work and it is good to show new data. **Changes in text** Fig-.2: The LEED pattern is now included as separate panel (e).

Q2: Given that FER spectra are highly sensitive to tip conditions and tunneling parameters, which are crucial for the LWF measurements, the authors should provide more detailed experimental parameters and conditions.

A2: The FER spectra have been measured in a bias window from 3-10 V at 0.1 nA setpoint current and enabled feedback loop. In Fig. 3, we show the differential-conductance (dI/dV) response measured with a lock-in amplifier during the bias sweep. The maximum in the dI/dV signal arises as the tip suddenly retracts from the surface when running through a FER, which in turn causes the dI/dV signal to increase temporarily. The maximum can thus be related to the energy position of the FER states. **Changes in text** p.8/top: Additional details on the measuring scheme of FERs.

Q3: Figures 4a and 4b appear to contain identical images, which should be corrected or clarified.

A3: While the topographic images in the left panels are indeed similar (only subject to a small lateral shift), the spectral data is complementary. In panel (a), the response of the tunneling current to a modulated tip-sample distance -d(lnI)/dz- is shown, while the current response to a bias modulation is displayed in panel (b). As the two measuring schemes cannot be realized simultaneously, two independent images had to be acquired, each one consisting of a topographic and a spectroscopic map. For the sake of completeness, all this data is presented in figure 4.

Reviewer 2:

Q1:. A large ‘strangeness’ of this STM field-emission-resonance (FER) spectroscopy method is this first peak, which has to be lowered by a ‘magic value’, here by 0.5 eV (page 4). In terms of WF, it already is a large value. The authors write ‘~0.5 eV’, so there seems to be an error. - I think that the authors have to justify this 0.5 eV value and to describe this phenomena of image-potential effects a bit more precisely. Is there a work that precisely describes this downshift? If so, please, cite this work. What is the error of the value and how does this influence the fits in Fig. 3e?
Note that in Fig. 3e, there are only 3 values after the first one concerning the sqrt(3) x sqrt(3) structure! The fitting therefore strongly depends on the first value and I guess that there easily is an error of +/- 1 eV for the WF of phi_Cr3O6/Pt(111) = 7 eV.

b) In view of a), please, mention an error for all the three exp. WF values (phi_Cr3O6/Pt(111), phi_Cr6O11/Pt(111), phi_Pt(111)).

c) Is the electric field, F, constant at all n’s? Please, justify.

A1: The FERs are typically described as electronic eigenstates of the triangular potential delimited by the sample surface and the vacuum energy that slopes down with the tip-electric field. While this approach is well justified for higher FER states, larger deviations are revealed for the 1st resonance, whose probability density is closest to the surface and whose energy is most affected by image-potential interactions. An electron in the lowest FER therefore induces a positive image charge in the Pt(111) surface, and the mutual attraction between the two leads to a downshift of the resonance energy of the order of □V = $-c/4z$ (with z being their separation). However, neither z nor the proportionality constant c can be determined precisely, as the spatial localization of the 1st FER and the screening ability of the oxide layer are unknown. There are two approaches to overcome this dilemma (i) ignore the 1st FER and perform the fitting only with higher states or (ii) artificially upshift the 1st FER to compensate for imagepotential effects. We have tested both schemes, as summarized in the newly added supporting information (SI).

Approach (i) leads to intermediated workfunction values, but fitting with sometimes only three maxima produces large error margins. Approach (ii) leads to more stable fitting routines, but requires the determination of a reliable offset value for the 1st FER. In our analysis, we determined this offset on the basis of results acquired on Pt(111), where the number of FERs is high and the impact of the unknown level offset is reduced. Optimizing the fitting for Pt(111) gave an offset of 0.5 V for the 1st FER that was consequently used for all other fitting procedures. To analyze the impact of this parameter, we have systematically changed the offset from 0 and 1.0 V, as displayed in the SI (Table 1). From the best fit performed with 0.5 V offset, we determined an approximate error of our workfunction analysis.

Note that approach (i) that ignores the 1st FER shows a much higher error margin of (□ ± 0.2 eV), as shown in table 1 of the SI as well.

The electric fields obtained from fitting Eq. 1 to experimental data have also been inserted into the SI. Not surprisingly, the values vary according to the fitting routine, however deviations only amount to ±15% for different offset values. For different sample systems, the fitted field strength in the tip-sample junction decreases with increasing thickness of the oxide layer, in correspondence to the dielectric nature of the oxide films on top of Pt(111) (see SI, table 2).

Changes in text:

p.5/top: Inserting error margins for all workfunction values as deduced from the fitting procedure

p.4/bottom: explaining more details of the fitting procedure, especially the origin of the energy offset of the 1st FER

SI: Adding a supporting information that shows in detail the impact of the different fitting routines (w/wo 1st FER, with different energy offsets of the 1st FER)

Q2: On page 4 it is suggested that effective barrier-height mapping yields a signal (d(lnI)/dz), which is somewhat proportional to sqrt(phi) on the surface. On the top of page 5 it is written that "… an approximate workfunction of 5.6 eV **for both**, the double-stack (2×2) film and bare Pt(111) (from Eq.1)" is found. In this case, I expect no contrast difference in d(lnI)/dz images between regions with the double-stack (2×2) film/Pt(111) and bare Pt(111) surface regions because those regions seem to have the same WF. However, this is not the case, see Fig. 4a (right): there is a clear contrast difference between the Pt(111) (bright) and Cr6O11/Pt(111) regions (dark). Why is this, please clarify.

A2: As mentioned in the text, the d(lnI)/dz data gives only relative information on the workfunction□ In fact, it probes modulations in the tunneling current as a function of the tip height, which can be connected to the apparent barrier height felt by the tunneling electrons. However, another central parameter affecting these current modulations is the spatial diffusivity of electronic states above the surface. Naturally, the free electrons in a metal surface are spatially more diffuse than the localized states of an oxide film, so that d(lnI)/dz measurements above Pt(111) give higher values than above the Cr_6O_{11} ad-layer. This effect only partly correlates to the macroscopic workfunction, explaining why d(lnI)/dz maps show a contrast between Pt(111) (brighter) and bi-stack Cr_6O_{11} patches (darker) despite similar □ values. We therefore use the d(lnI)/dz data only for qualitative purposes and do not aim to derive quantitative □ values from this data.

We note in passing that there is a workfunction difference between Pt(111) and Cr_6O_{11}/Pt(111), both in the calculations and the FER spectra, in agreement with the detected contrast in d(lnI)/dz maps (Fig. 4a).

Q3: The term "workfunction" (WF) is not really well defined in this work, in particular not in the experimental section. What do the authors actually measure in the experiment (and calculate in the theory)? I guess that the WF is given by the film **and** the underlying support and not the film alone. However, in many places the reader gets the impression that only the film is meant, which would be strange. The entire manuscript has to be revised that potential misunderstandings do not appear. Suggestion: just define and clarify the WF somewhere at the beginning of the manuscript by also introducing phi_Cr3O6/Pt(111), phi_Cr6O11/Pt(111) and phi_Pt(111).

A3: The referee is perfectly correct that the given workfunction values always concern the combined oxide/support system, especially because the films are by far too thin to develop their own bulk-type workfunction response. We have clarified this point in the revised version of the manuscript.

Changes in text: Stating at several points (e.g. p4, bottom) that measured workfunction values always concern the combined metal/oxide system

Q4: Because phi_Cr3O6/Pt(111) is from the film and the Pt(111) support and because Pt(111) already has a very high WF (5.9 eV !) with respect to many other (111) surfaces, even compared to Pd(111), I have the feeling that the high value of phi_Cr3O6/Pt(111) is somewhat also due to the WF of the Pt(111) surface. If one places, in a 'Gedankenexperiment', the same film onto, e.g., Ag(111), the WF should be way much smaller because the WF of Ag(111) is much smaller (4.53 eV!). Is this the case and can this be stated like this?

A4: The question on the workfunction of Ag-supported CrO_2 trilayers is indeed interesting and we have performed respective calculations using the same computational setup. The results show a somewhat larger workfunction increase upon trilayer deposition on Ag(111) as compared to Pt(111). The resulting overall workfunction of the CrO_2/Ag system remains however lower than that of CrO_2/Pt, because of the reduced initial workfunction of bare Ag(111). The overall concept, in which interfacial charge transfer governs the workfunction of a metal/oxide system, remains however valid also in case of the Ag(111) support. At present, our results on CrO_2/Ag(111) still have preliminary character and we therefore refrain from adding them to the manuscript.

Q5: The authors mention a WF value of phi_Pt(111) = 5.7 eV for the Pt(111) surface on page 6 (line 25). a) I guess that this is the author's calculated value. Is this correct?

b) To my opinion WF values should be always compared with literature values. So, how does the exp. and theo. value of 5.7 eV compare with literature values? The authors could use references [1] and [2]: [1] Derry, G. N. and Kern, M. E. and Worth, E. H., Recommended values of clean metal surface work functions. J. Vac. Sci. Technol. A 33, 060801 (2015).[2] Kawano, H., Effective Work Functions of the Elements. Appl. Surf. Sci. 97, 100583 (2022).c) Are there any WF values previously measured on the Cr-oxide films ? How do compare those values with the ones obtained here?

A5: We follow the reviewer's advice and have complemented the calculated □ value for Pt(111) (6.1 eV) with measured ones. For this purpose, two references have been added to our manuscript:

[27]: Kawano, H. Effective Work Functions of the Elements. *Prog. Surf. Sci.* **2022**, *97*, 100583 ❼ $\square_{Pt(111)}$ = 5.7 eV

[28]: Derry, G.N.; Kern M. E.; Worth, E. H. Recommended values of clean metal surface work functions. *J. Vac. Sci. Technol. A* **2015**, *33*, 060801 ❼ 5.7 eV $< \square_{Pt(111)} <$ 6.1 eV

Q6: Page 8: many details are clearly missing in this experimental method section. I consider it incomplete. If more space is needed the authors could use a supporting information document. Please, consider:

A6: We have added missing information to the experimental section of our manuscript.

Changes in text (p.8):

- STM: Pan-type, Createc
- E-beam evaporator: Tectra
- Crystal: Pt(111)
- Ad-metal: Cr (99.9%)
- Gases: Oxygen (99.998%), chamber backfilling
- Sputtering: Ar at 1500 eV, 5μA ion flux, 1h
- z-modulation for d(lnI)/dz imaging: 0.5 Å
- V-modulation for dI/dV spectroscopy: 20 mV
- Fig 4a shows the d(lnI)/dz contrast

Q7: The authors should mention in the abstract and introduction that STM (spectroscopy) was done at 100 K.

A7: The temperature of the experiments has been added directly to the abstract.

Q8: Page 2 with "The underlying mechanisms are often inaccessible to direct measurements and can be retrieved only by theoretical means." Please, check the meaning of the sentence. **A8:** The content of this sentence becomes clear only in combination with the preceding sentence that mentions the unique electronic character of each layer of a multi-stack oxide film. This layer-wise electronic variability cannot be probed directly but only its collective response shows up in an experiment, for instance as workfunction or adsorption characteristic.

Q9: Page 2 with "In fact, most experimental techniques are sensitive to global quantities only, such as the mean workfunction that arises from the electron distribution across all layers of the oxide stack." To my personal opinion, this sounds a bit too negative. There were in the past quite a few SPM works that could reveal and explain WF phenomena at the nm scale. Think about all the Kelvin probe force microscopy (KPFM) work done on oxide thin films. With respect to STM, there is much less, I agree. – In any case it would be interesting to see whether or not KPFM gives the same results as STS.

A9: We fully agree with the reviewer at this point, as KPFM has proven to be a powerful technique to measure local workfunction trends on surfaces. To overcome this shortcoming, the KPFM method plus respective citation are now explicitly mentioned in the outlook of our work.

Changes in text (p.8):

Providing a reference to KPFM as powerful means to probe local workfunction modulations and add citation [33]: Barth, C.; Foster, A.S.; Henry, C.R.; and Shluger, A.L. Recent Trends in Surface Characterization and Chemistry with High-Resolution Scanning Force Methods. *Adv. Mat.* **2011,** *23*, 477-501.

Q10: Figure 2: Why is the quality of the LEED image so low? Do the authors have another image with a higher quality?

A10: Thank you for the comment. We have replaced the LEED image with better data.

Q11: Page 6, line 26: "(phiCr3O6 = 8.0 eV and phiCr6O11 = 5.7 eV for)" <= What is coming after 'for ...' ?

A11: The misspelling has been corrected.

Q12: Page 7, line 48: "as discussed Ref. [21]" => "as discussed **in** Ref. [21]" **A12:** The misspelling has been corrected.

Reviewer 3:

Q1: Equation 1 appears incomplete. It should be written as (n−1/4)2/3. Note that for n≫1, (n−1/4)2/3≈n2/3. The extrapolation of the linear fitting function of Un versus n2/3 for higher-order FER states toward n=0 should provide a reasonable estimate of the work function. For clarification, please refer to Equation 2 in this reference. DOI 10.1088/1361-648X/aba3f0

A1: We agree with the referee that we should not rely on the simplified formula for high n values: $E_n \sim n^{2/3}$, but include the corrected n-term: $E_n \sim (n\text{-}1/4)^{2/3}$. With this, we follow the seminal paper of Gundlach and the reference proposed by the referee that have both been added to our manuscript. The use of the corrected dependence $(n\text{-}1/4)^{2/3}$ leads to some modifications in the fitted workfunction, however, the outstandingly high value of $\square_{Cr6O11}$ ~ 7.1 eV and the workfunction ordering of $\square_{Cr3O6} > \square_{Pt} > \square_{Cr6O11}$ is not affected by the refined fitting procedure.

Changes in text:

(p.4): Correction of equation 1 and in the corresponding workfunction

fits **New references:**

[24] Gundlach, K. H. Zur Berechnung des Tunnelstroms durch eine trapezförmige Potentialstufe. *Sol. Stat. Electr*. **1966**, *9*, 949-957.

[25] Aladyshkin, A. Y. Quantum-well and modified image-potential states in thin Pb(111) films. *J. Phys.: Condens. Matter* **2020**, *32*, 435001.

Q2: For Fig. 1b, Fig. 1c, Fig. 2b, Fig. 2c, and Fig. 2d, please include scale bars on the STM images or clearly indicate the size of each image.

A2: The dimensions of the STM images are now given in the respective figure captions.

Q3: On page 4, the text states, "The 1st FER is typically downshifted by ~0.5 eV due to image-potential effects in the tip-sample gap." Please provide a reference to support this claim if it is meant as a general observation for all FER measurements.

A3: A similar question has been posed by referee 2 (question Q1). We kindly ask you to check our answer there and the comprehensive discussion on how to determine the energy offset of the 1st FER in the supporting information (SI).

Changes in text:

SI: Adding a supporting information that shows in detail the impact of the different fitting procedures (w/wo 1st FER, with different energy offsets of the 1st FER)

Q4: In the apparent barrier height map spectroscopy, the modulation amplitude is not specified. Please indicate either the applied AC voltage or the amplitude of tip displacement used in the measurement.

A4: We employed a z-modulation amplitude of 0.5 Å for the acquisition of d(lnI)/dz images.

Changes in text: The modulation value is now state in the experimental section on p.8.

Q5: In Fig. 3e, five data points are shown for the (2×2) system. However, Fig. 3c displays six FER peaks. Please clarify why one data point appears to be omitted.

A5: We apologize for this mistake and have carefully rechecked the content of Fig. 3e. The displayed data in Fig. 3a-d and 3e is now equivalent in every detail.

Changes in text: Revision of figure 3e with all mistakes corrected.

Q6: In Fig. 3e, no data points are reported for the FERs corresponding to defect (as shown in part b). Please address this omission or include that as well.

A6: A qualitative workfunction analysis of defects in the (□3×□3)R30° patches in Fig. 3e has been omitted for two reasons: (i) The local nature of the oxide defects is highly variable, which results in a large spread of the associated workfunction values. The example shown in Fig. 3b shall only demonstrate the general trend, namely the drastic workfunction decrease in presence of defects. (ii) We do not want to overload the diagram in Fig. 3e, as the defect data would lie almost on top of the ones for the (2×2) oxide patches and the bare Pt(111).

Q7: In Fig. 3e, the reported resonance energies (y-axis) do not align with the FER peak positions in the corresponding dI/dV spectra. For example, for the (2×2) and Pt(111) systems, Fig. 3e shows the first FER at ~6.8 V, whereas in the dI/dV spectra, the first FER peaks appear around ~5 V. Please explain or correct this discrepancy.

A7: We apologize for this mistake and have carefully rechecked the content of Fig. 3e. The displayed data in Fig. 3a-d and 3e are now equivalent in every detail.

Changes in text: Revision of figure 3e with all mistakes corrected.

Q8: In Fig. 3e, the x-axis should be n2/3. As it was mentioned in the second bullet point, extrapolation of the linear fitting function for the dependence Un on n2/3 for the higher-order FER states should be taken into account. Please check Fig. 6 in this paper for reference

A8: We would prefer to show n and not $(n\text{-}1/4)^{2/3}$-values on the x-axis. This has the advantage that FERs measured in the spectra can directly be assigned to points in the diagram. However, the respective fits do not appear as straight lines anymore but have a curved shape. While both presentations come with their own advantages, we would like to go with the first option that is showing the FER number n on the x-axis.

Q9: On page 6, the text states that based on the apparent barrier height map, WF Cr3O6> WF Pt ~ WFCr6O11. If this is accurate, why isn't a similar or comparable contrast observed between Pt and Cr_6O_{11} in the d(ln I)/dz map in Fig. 4? That is, why is there a visible contrast between Pt and Cr_6O_{11} in the ABH map? Please also include a color scale bar next to the map for clarity.

A9: The refined fitting procedure of the FERs based on the $(n-1/4)^{2/3}$ term indeed leads to a workfunction difference between the Cr_6O_{11} film and bare Pt(111). This difference is also in line with literature values that find a systematically higher workfunction of Pt(111) (up to 6.1 eV) compared to the calculated value for Cr_6O_{11}/Pt(111). The d(lnI)/dz contrast between the (2×2) oxide and the bare Pt can thus be rationalized already from the reported workfunction values.

However, another parameter, affecting the experimental contrast, is the spatial diffusivity of electronic states above the surface. Naturally, the free-electron like states above a metal surface are spatially more diffuse than the localized states of an oxide film, so that d(lnI)/dz measurements on Pt(111) give higher values than on Cr_6O_{11}/Pt(111). This effect only partly correlates to the macroscopic workfunction and explains, why d(lnI)/dz maps

show a clear contrast between Pt(111) (brighter) and bi-stack Cr_6O_{11} islands (darker) despite similar values. Note that we use the d(lnI)/dz data only for qualitative purposes and do not aim to derive quantitative values from this data.

Changes in text: Fig. 4: Scale bares have been added to the spectroscopic maps.

Q10: For Fig. 5b, if the plot shows the projected (calculated) density of states, it would be clearer to label the y-axis as PDOS rather than LDOS, or to mention in the caption that this represents the calculated density of states.

A10: We have followed the suggestion of the referee and specified that the y-axis in Fig. 5b displays the calculated local densities of states (LDOS) of the system.
